# Facilitators and Challenges to Start-Up of the Colorectal Cancer Screening Demonstration Program

**Published:** 2008-03-15

**Authors:** Amy DeGroff, Jennifer Boehm, Laura C Seeff, Sonya Goode Green, Debra Holden

**Affiliations:** Centers for Disease Control and Prevention; Division of Cancer Prevention and Control, National Center for Chronic Disease Prevention and Health Promotion, Centers for Disease Control and Prevention; Division of Cancer Prevention and Control, National Center for Chronic Disease Prevention and Health Promotion, Centers for Disease Control and Prevention; Research Triangle Institute, Inc, Research Triangle Park, North Carolina; Research Triangle Institute, Inc, Research Triangle Park, North Carolina

## Abstract

**Introduction:**

The Centers for Disease Control and Prevention (CDC) funded the Colorectal Cancer Screening Demonstration Program in 2005. To assess the feasibility of providing community-based colorectal cancer screening, CDC is conducting a multiple-case study as part of a larger evaluation effort. This article highlights key facilitators and challenges common to the five programs studied during the start-up period.

**Methods:**

The multiple-case study that includes all five program sites is being conducted during the 3-year program as part of process evaluation efforts. Data collection for program start-up occurred during August 2005 through September 2006. Data include approximately 70 interviews with program staff and stakeholders, document review, and observations. Both case-specific and cross-case analyses were conducted.

**Results:**

On the basis of the cross-case analysis, we identified four factors that facilitated program start-up and four factors that challenged program start-up. Facilitating factors included 1) pre-existing program infrastructure, 2) partnerships, 3) clinical expertise, and 4) program champions. Factors challenging program start-up included 1) contracts with endoscopists, 2) resources for treating medical complications of screening and for cancer treatment, 3) administrative barriers, and 4) resource limitations. Additionally, preplanning was critical, allowing programs to efficiently initiate activities once funds became available.

**Conclusion:**

The most important facilitator identified was the ability to build on pre-existing infrastructure, which provided experienced staff, partnerships, and provider relationships, as well as aided program integration with other chronic disease programs. Results also suggest that substantial planning and partnership development can begin before funds are secured to implement a colorectal cancer screening program.

## Introduction

Colorectal cancer is the third most common cancer among both men and women in the United States (1). Although evidence suggests that regular colorectal cancer screening reduces colorectal cancer incidence and mortality (2), few organized, population-level screening programs have been implemented and evaluated in the United States. Consequently, the Centers for Disease Control and Prevention (CDC) funded five Colorectal Cancer Screening Demonstration Program (CRCSDP) sites in 2005 for a 3-year period (3).

To assess the feasibility of providing community-based colorectal cancer screening, CDC is conducting a multiple-case study (4,5) as part of a larger evaluation effort. Program evaluation is a critical function of public health, and demand continues to grow for evaluation to assess program implementation and effectiveness (6). The purpose of the case study is to evaluate implementation of the CRCSDP and accurately describe how the program was carried out — that is, to understand *what happened* (7). Case study results are likely to offer valuable insights to others throughout the United States who are beginning to plan colorectal cancer screening programs.

We present case study findings for the start-up period, the time between initial program funding and initiation of screening services. Activities conducted during the start-up phase of the CRCSDP included hiring or assigning staff; assembling medical advisory boards (MABs); developing program models, policies, and procedures; enlisting partners; developing data collection and reporting systems; planning client recruitment strategies; and identifying resources for treating complications. A description of individual program models and start-up activities are detailed elsewhere (8). This manuscript presents results of a cross-case analysis and identifies the key facilitators and challenges to the start-up of CRCSDP sites.

## Methods

We conducted a multiple-case study (4,5) during the program start-up period as part of the process evaluation efforts. All CRCSDP sites were included in the case study, representing five unique "cases." Participants included grantee staff and stakeholders involved in program start-up and CDC staff providing program oversight and technical assistance. Data were collected during August 2005 through September 2006 and included document review, approximately 70 interviews, and field observations. Atlas.ti (Atlas.ti Scientific Software Development GmbH, Berlin, Germany), a software program for qualitative data, was used to facilitate analysis. A codebook with detailed code definitions was developed, and standard content analysis (9) was used to make inferences from the data.

Given the multiple-case study design, we conducted both case-specific and cross-case analyses (4,5,10). To understand each unique case, first we conducted individual case analysis (4,5). Cross-case analysis then was carried out to identify findings common across the five cases (4,5,10). A detailed account of the methodology is summarized elsewhere (8).

## Results

On the basis of the cross-case analysis, we identified four factors that facilitated program start-up and four factors that challenged program start-up. Facilitating factors were 1) pre-existing program infrastructure, 2) partnerships, 3) clinical expertise, and 4) program champions. Challenges to program start-up were 1) contracts with endoscopists, 2) resources for treating medical complications and for cancer treatment, 3) administrative barriers, and 4) resource limitations.

### Facilitators to program start-up

**Pre-existing program infrastructure**

The most important facilitator of program start-up was the use of pre-existing health program infrastructure, on which CRCSDP sites built to develop their colorectal cancer screening programs. Sources of infrastructure included the National Breast and Cervical Cancer Early Detection Program (NBCCEDP), the Well-Integrated Screening and Evaluation for Women Across the Nation (WISEWOMAN) program, other health initiatives such as prostate cancer screening programs, colorectal cancer screening programs in other parts of the states, previous colorectal cancer screening pilot programs, previous research assessments of colorectal cancer screening capacity, and structures and systems within the grantee institution itself (Table 1). NBCCEDP and the  WISEWOMAN program are other CDC-funded screening programs for breast and cervical cancer and cardiovascular health, respectively (11,12). Some of these efforts provided opportunities to gain experience in implementing public health screening programs, build relationships with providers, and test screening models well in advance of participating in the CRCSDP. All contributed to the readiness of each site to initiate the new CRCSDP.

The NBCCEDP was identified as an important framework for program planning in four of the CRCSDP sites by offering structure, systems, and experience. The fifth site did not have experience with the NBCCEDP. The CRCSDP sites typically used components of the NBCCEDP model (e.g., provider system, data management system) and modified them to fit their new model for colorectal cancer screening. For instance, sites used their pre-existing NBCCEDP network of clinical providers to deliver screening services (e.g., distribute and process fecal occult blood test kits) and make referrals for colonoscopy. The sites also recruited MAB members from the NBCCEDP to establish a new MAB for the CRCSDP, they integrated colorectal cancer-related data management and billing into existing NBCCEDP systems, and they planned to recruit clients for colorectal cancer screening through "in-reach" (i.e., recruitment of clients of an existing program). One stakeholder working with a provider site said, "When it came time to start policies, procedures, contracts, I think I had a leg up on that because of doing all of that with [NBCCEDP]."

**Partnerships**

Partnerships emerged as another important facilitator of program start-up, yielding benefits including technical support, expertise, and resources. Most important among partners were the Comprehensive Cancer Control (CCC) programs in the states with CRCSDP sites. CCC activities typically are coordinated by the state health department and engage a wide range of stakeholders to develop and implement statewide cancer control plans. In some cases, the CCC programs had planned well in advance of the CRCSDP initiative and provided important support during the start-up period. A unique contribution of the CCC programs involved brokering access to a network of well-established relationships, such as with leading colorectal cancer experts in the state who could be recruited for participation on the MAB. In addition, individual members of the CCC programs were ready to support and advocate for the new colorectal cancer screening programs. One staff member said, "I don't know if we could have had some of the doors open as wide and as quickly if we didn't have the comp cancer program." In addition, CCC groups provided instrumental in-kind staff support and financial resources to the CRCSDP sites.

For two of the sites, CCC programs played a central role in applying for CRCSDP funds from CDC. One person noted, "The [colorectal cancer task force of the CCC program] had come up with its own mission statement and vision, so there had been a lot of team building before the writing of the demo grant. It was a good foundation in order for us to move forward." In addition, some of the CCC groups had been awarded competitive funds to host a Dialogue for Action, a day-long event sponsored by the Prevent Cancer Foundation and CDC to increase colorectal cancer screening by convening providers, medical specialists, researchers, and representatives of government and nongovernment agencies. These events had helped to mobilize providers, many of whom were later invited to participate in the CRCSDP.

Other key partners included the American Cancer Society (ACS), local universities, and cancer treatment centers (Table 2). In one site, ACS provided important professional education materials to participating clinical provider sites. In another site, a local university helped to develop data collection forms and plan an evaluation of recruitment activities.

**Clinical expertise**

Interviewees emphasized the value of having clinical expertise available early in the start-up process. Staff members suggested that the CRCSDP was more medically complex than the NBCCEDP, which several program directors also managed. Clinical expertise came primarily from two sources: program staff and members of MABs. Individuals with expertise in the delivery of colorectal cancer screening services assisted in developing program policies, patient flow processes, data collection systems and related forms, treatment plans, and quality assurance measures.

One staff member acknowledged the challenges of initiating the CRCSDP without access to in-house staff possessing clinical knowledge:

I felt like there needs to be more clinical expertise in the department. Because we didn't have that, we really struggled. . . . [We] had difficulty understanding the various protocols associated with the tests and communicating with physicians and clinicians . . . so I think, would this program start over again, I would say you really need to have a clinical lead with expertise and background in this area.

Others believed their staff with clinical training lent credibility to the new program and facilitated communication between the program and its MAB, as well as participating providers. One person said:

I think you do need a clinical person. I don't know that it has to be a physician, but I think you at least have to have a nurse to take a look to see that things are making sense. I also think that's true when you're talking with providers in terms of bringing them on board — they need to understand that you really know what you're talking about.

**Program champions**

Program champions were another facilitator of program start-up. Although champions were identified at various levels, the program directors often were viewed as the overall champion during start-up. Their understanding of the whole initiative, their ability to build relationships, and their reputation for past successes led others to want to be involved with the new CRCSDP. One physician on an MAB said, "I've worked with [the program director] previously . . . so she was a major reason for my participating on this project."

Champions were identified at other levels of the program as well. In several cases, programs engaged leading gastroenterologists in their state or region to participate in their MAB; these physicians often became champions for the program during the start-up period, offering legitimacy for the CRCSDP in the medical community. One staff member noted, "If other doctors hear that [MAB member] has okay'd something, or that he agrees with it, then they are okay with it because of his reputation and history of involvement with cancer issues."

Champions also existed at individual provider sites, negotiating the bureaucracy of their own institutions to facilitate integration of the CRCSDP into their systems and build support for its implementation. One physician, acknowledging this role, remarked, "I see myself as a consultant, a colon and rectal surgeon who knows the problem, who knows what surgeons do, what gastroenterologists do, what patients do, and what hard-headed institutions do who don't want to yield." These champions all exhibited important leadership and commitment toward ensuring the successful start-up of the CRCSDP.

### Challenges to program start-up

**Contracts with endoscopists**

Contracting endoscopists to conduct colonoscopies proved to be a challenge for some sites. Staff and stakeholders identified the following barriers to endoscopists' participation: 1) burdensome government contracting processes; 2) ethical, financial, and liability concerns about the lack of treatment for people diagnosed with cancer, even though the individual programs had identified sources for treatment; 3) capacity limitations among providers; 4) Medicare reimbursement rates being seen as low compared with other insurance sources; 5) data and paperwork requirements for the CRCSDP; 6) overall disruption to the endoscopist's practice in integrating a small number of CRCSDP clients; and 7) concerns about poor compliance (e.g., no-shows, inadequate bowel preparation) in a low-income client population.

One staff member acknowledged that, overall, endoscopists or their institutions might perceive the program to be too burdensome:

It's sort of a balancing act . . . even with concerned physicians [gastrointestinal specialists] who are interested and want to do this systematic approach. It may not make sense when you look at it from the business manager's side, or paperwork demands. The [NBCCEDP] program disrupts every aspect of a program from medical people, billing staff, contract people, all of them — our system disrupts their systems. It's not really value added for them; there's no advantage for them.

On the basis of their start-up experience, interviewees recognized the need to initiate relationships with endoscopists early on, engaging them in the planning process from the start. One physician suggested, "You need to find somebody [i.e., an endoscopist] who has the passion to understand the importance of screening and understands that it will cost them time that they may not be paid for, but that it's the right thing to do."

**Resources for treating medical complications and for cancer treatment**

Consistent with the NBCCEDP, CRCSDP funds could not be used to pay for cancer treatment, and CDC required that sites applying to participate in the CRCSDP secure treatment resources as a condition of funding (13). Similarly, CDC funds could not be used to support the costs of treating unintended medical complications resulting from screening examinations. Although resources for treatment had been identified before programs were selected, in many instances these resources were too limited or not reliable, raising both ethical and practical concerns. Program staff and stakeholders expressed apprehension about screening a population that could not pay for treatment and might not qualify for assistance. One staff member said, "There's nothing more distressing for us or clinicians than to know that you have someone with positive results [cancer], but there is no funding mechanism available for treatment."

Sites also faced the practical challenge of requesting no-cost treatment services from local cancer centers and hospitals or considering how best to facilitate clients' applications for emergency Medicaid or other financial assistance. One staff member noted, "[Securing resources is] very 'piecemeal.' . . . We've had problems in our other program where we couldn't get a patient on emergency care, and having to piece together surgery, chemo, pharmacy support, etc., is incredibly difficult." In more rural settings, there were concerns that cancer centers in the largest cities would shoulder the burden for treatment. The lack of treatment resources also deterred some providers from participation in the CRCSDP, given their own ethical concerns, as well as worries about a potential financial burden for their practice or institution.

To address this challenge, program staff emphasized the importance of developing partnerships with potential sources of cancer treatment, including cancer centers and hospital-based oncology departments. In several instances, MAB members facilitated these relationships. In addition, staff members suggested the need to identify more than one treatment provider to "share the burden."

**Administrative barriers**

Administrative challenges such as hiring and retaining staff, establishing contracts, and developing and implementing data and billing systems led to delays in the start-up process. In one case, pay scales were blamed for the inability to hire a nurse coordinator and forced the program to alter its program model more than 2 months into the start-up period. In another, staff turnover in a key position left remaining staff to fill the void during a time-consuming hiring process. Contracting proved problematic for several CRCSDP sites, particularly for providers who had not worked with public agencies in the past. One staff member said, "Establishing new contracts with people we've never worked with before is more challenging. The [agency contract] is long and can shock potential contractors." Finally, developing the data management system and integrating it into existing service delivery systems was time consuming and challenging, and in some settings, the reporting requirements were perceived as burdensome. "We're coming to [the providers] saying, 'For our 20 patients, can you give us a paper bill, fill out this special form, use a different appointment system than what you normally do, and by the way, can you also sit on our MAB?' "

**Resource limitations**

The final challenge to program start-up involved resource limitations. Given expectations that programs would implement screening at 6 months, the CRCSDP sites allocated a substantial portion of their first-year budget to screening costs and made difficult compromises in staffing and other program components. This left some sites relying primarily on existing staff (i.e., in-kind) supported by other program funds and with other responsibilities. One staff person said, "The CCC has basically evolved in supporting the CRC program for the past 2 years. If we hadn't had that ability to move staffing responsibilities around, I'm not sure we would have wanted to start the project." Another staff person emphasized the importance of in-kind contributions: "If CDC gets all this information back and thinks that what we're actually spending to make this program work is what it would actually cost, then they're not in line. . . . I was surprised though with what we were able to accomplish with the funding we got." On the basis of the start-up experience, staff members suggested that financial resources should be obligated to support key coordinating positions for the program.

## Discussion

We identified several important facilitators for program start-up, including using pre-existing infrastructure, building partnerships, engaging clinical expertise, and involving strong champions. Of these, the use of pre-existing infrastructure was the most valuable in advancing the start-up of the CRCSDP. Attempts to integrate the CRCSDP with other chronic disease programs such as the NBCCEDP, WISEWOMAN, and other screening programs are consistent with broader calls in public health to move from "silo-funded" categorical programs to a more integrated model (14). This integration has the potential to increase efficiencies in staff, funds, and interventions (14). Most importantly, clients served through these types of integrated programs may benefit from receiving a comprehensive array of screening services.

Results also suggest that critical planning for colorectal cancer screening and related partnership building had been conducted by sites even before the CDC funding opportunity arose. This work seemed to position the programs to compete successfully for the CRCSDP award and to move more quickly through the start-up period to initiate screening. These results suggest that even for states with minimal or no funding yet available for colorectal cancer screening, important planning and partnership development can be accomplished to prepare for future opportunities. Once funding is secured, such efforts are likely to both shorten the time needed for program start-up and support its success. Furthermore, programs can build partnerships with clinical experts and potentially diminish challenges related to contracting with endoscopists if relationships with these providers can be established early in the planning process and their support enlisted.

Champions emerged as important in negotiating the multi-organizational character of the CRCSDP service delivery environment. Given the full continuum of screening and diagnostic services (e.g., client recruitment, assessment, screening, patient navigation, cancer treatment) offered through the CRCSDP, sites developed program models that involve a networked system of agencies or departments, each providing a unique service or services within that continuum (8). This type of interagency collaboration to support the CRCSDP is increasingly recognized as important in the broader implementation of public health programs (15). The strong facilitative skills of the directors and coordinators of these colorectal cancer screening programs were essential to effectively engage stakeholders, build partnerships, negotiate common goals, and produce a well-designed program. These skills have been recognized in the public management and public health literature as increasingly important to successful program management (16-18).

Our findings also highlight concerns about securing resources for treating unanticipated complications of screening and for treating cancer in an uninsured and underinsured population. The NBCCEDP experienced similar challenges before the National Breast and Cervical Cancer Prevention and Treatment Act of 2000 (19); those programs largely succeeded in securing treatment for women diagnosed with breast and cervical cancer, but not without substantial difficulty (20). A more permanent solution to securing treatment resources would benefit an expanded colorectal cancer screening program.

Finally, results suggest that reliance on staff with competing programmatic responsibilities may further challenge program start-up. Given the intensive effort required to develop the CRCSDP, allocating adequate resources for dedicated staff is important. This may be especially crucial given the clinical complexity of the program, the involvement of multiple provider specialties, and the comprehensive range of services provided (e.g., public education, outreach, screening, patient navigation).

Although these findings represent only five sites, the identified facilitators and challenges to program start-up provide important information for other emerging programs. The facilitators were common across all CRCSDP sites in supporting the start-up of the CRCSDP, and a new program is likely to struggle without attending to each. In contrast, some of the challenges (e.g., contracts with endoscopists, administrative barriers) are more likely to be site-specific, depending on the unique characteristics of the program and its context, and others (e.g., resources for treatment) may reflect system-level challenges. The formative evaluation conducted through this case study is yielding results that provide information for ongoing program improvement and for more widespread screening efforts in the future. Subsequent reports will provide evaluation findings from the screening implementation phase of the CRCSDP.

## Figures and Tables

**Table 1:** Sources and Contributions of Pre-existing Infrastructure Used by the Colorectal Cancer Screening Demonstration Program, 2005–2006

Source of Infrastructure	Contributions of Infrastructure
National Breast and Cervical Cancer Early Detection Program (NBCCEDP) and Well-Integrated Screening and Evaluation for Women Across the Nation (WISEWOMAN)	Network of clinical providers
	Data management and billing systems
	Client base for colorectal cancer screening
	Case management system
	Medical advisory boards
	Public education materials
	Staff experience and capacity
Other health or screening initiatives	Staff experience and capacity
	Client base for colorectal cancer screening
	Provider relationships
Other colorectal cancer screening programs in the state, previous colorectal cancer screening pilots, or research	Staff experience and capacity
	Assessment and evaluation data
	Screening model
	Medical advisory boards
	Cancer treatment resources
	Policies and procedures
	Patient referral systems
Grantee institution	Clinical expertise
	Network of clinical providers
	Cancer treatment resources

**Table 2:** Partner Involvement in Start-Up of the Colorectal Cancer Screening Demonstration Program, 2005–2006

Partner	Activities
Comprehensive cancer control programs	Planning, advocating, and identifying medical advisory board members; brokering partnerships; hosting Dialogue for Action^a^; and providing in-kind and financial resources
Colorectal cancer task forces	Planning, grant writing, and advocacy
American Cancer Society	Identifying medical advisory board members; providing educational materials; and conducting public education campaigns, outreach efforts, and client recruitment
Local universities	Developing data collection forms and public education materials and conducting evaluations
Cancer treatment centers	Providing cancer treatment services

a A day-long event sponsored by the Prevent Cancer Foundation and Centers for Disease Control and Prevention to increase colorectal cancer screening by convening providers, medical specialists, researchers, and representatives of government and nongovernment agencies.
